# Pattern of Dermatological Disease Encountered in a Hematology Ward: A Retrospective Analysis of Dermatology Consultation in a Hematology Ward in a Tertiary Care Center in Saudi Arabia

**DOI:** 10.1155/2019/9891270

**Published:** 2019-01-13

**Authors:** Amal Aboud Alasmari, Anadel Hassan Hakeem, Fatemah Saleh Bin Saleh, Shahad Yousef Alsaigh, Waleed Al Ajroush, Laila Ali Layqah, Salim Alawi Baharoon

**Affiliations:** ^1^College of Medicine, King Saud Bin Abdulaziz University for Health Sciences, Riyadh, Saudi Arabia; ^2^Department of Dermatology, King Abdulaziz Medical City (KAMC), National Guard Health Affairs (NGHA), Riyadh, Saudi Arabia; ^3^Research Coordinator, King Abdullah International Medical Research Center, Riyadh, Saudi Arabia; ^4^Intermediate Care Unit, King Abdulaziz Medical City (KAMC), National Guard Health Affairs (NGHA), Riyadh, Saudi Arabia

## Abstract

*Introduction. *Skin manifestations are common in hematology ward patients and can result from infection, malignancy, or chemotherapy. The purpose of this study was to identify the most common dermatological problems encountered in the adult hematology ward at King Abdullah Specialist Children Hospital (KASCH).* Methods. *This was retrospective chart review of 78 dermatology consultations based on electronic health records for all inpatients in hematology wards at KASCH between January 2016 and December 2017. Data were presented as mean ± SD for continuous variables.* Results. *During the study period, a total of 1391 inpatients were referred to the dermatology department. A total of 403 (29.0%) referrals were from the internal medicine department and 78 (5.6%) were from the hematology department, six of which were rejected by the dermatology department. Almost all requests for referral were managed on the same or the next day with only two requests after 3 days. There were more female (n = 40; 51.3%) than male patients (n = 38; 48.7%) and the average age ± SD was 40.7 ± 19.8 years. Patients were diagnosed with a diverse range of hematological diseases. A total of 27 (35.1%) patients were diagnosed with acute myeloid leukemia. Overall, 98 differential diagnoses were made by dermatologists with only 26 being confirmed by skin biopsy. Eight (30.8%) patients were diagnosed with graft versus host disease confirmed by skin biopsy. The diagnoses were changed in 12 cases after skin biopsy. Several types of dermatitis were diagnosed in hematology ward patients including stasis dermatitis and contact dermatitis. The source of infection was not specified in most cases and the infection was treated empirically.* Conclusion. *Various dermatological disorders and cutaneous manifestations are observed in hematology inpatients with morbilliform drug eruption and graft versus host disease being the most common.

## 1. Background

Dermatology is primarily an outpatient-based service; however dermatologists play an integral role in the care of inpatients in multidisciplinary teams. The diagnosis and treatment of skin problems in inpatients are usually performed by non-dermatologists [1]. Previous studies have shown that about 80% of skin diseases in inpatients are misdiagnosed by non-dermatologists [1–6]. Furthermore, dermatologists need to change treatments in inpatients for their skin diseases in 60% of cases, which highlights the importance of providing further education for non-dermatologists regarding some common skin conditions [2, 4, 5]. Several studies reported that, in cases in which dermatological consultation was available, most dermatological referrals were received from the internal medicine department [3, 4, 7–9].

Hematology referrals constitute a significant proportion of consultations [3, 4, 7, 9–11]. Dermatological manifestations are common in hematology ward patients and can result from infection, malignancy, or chemotherapy [10, 11]. In a retrospective study conducted over 6 months in 2010, Koh found that 8.3% of all dermatology consultations came from hematology wards [11]. Cutaneous infections accounted for 15% of all dermatological diagnoses followed by cutaneous adverse drug reactions and dermatitis (17% and 13%, respectively) [11]. Herpes labialis was the most commonly encountered cutaneous infection [11]. In a recent study conducted in Dammam, Saudi Arabia, the authors reported that the most common skin features in hematology outpatient clinics were diffuse alopecia (66%), pallor (51%), skin dryness (35%), pruritus (23%), and hair thinning (20%) [10]. In a 1-year cross-sectional study conducted at the BSMM University, some dermatological manifestations were found to be more common in specific groups of patients than others [12]. For example, malignant infiltration and hemorrhagic findings were more common in acute lymphoblastic leukemia (ALL) patients whereas gingival hyperplasia was only diagnosed in acute myeloid leukemia (AML) patients [12].

The aim of the present study was to identify the most common dermatological problems encountered in the adult hematology ward of King Abdullah Specialist Children Hospital (KASCH) to provide adequate awareness of these conditions for non-dermatologists.

## 2. Methods

This was retrospective chart review of 78 dermatology consultations based on electronic health records for all inpatients in hematology wards at KASCH over a period of 2 years between January 2016 and December 2017. Data included in the analysis were age, gender, date of referral request, reason for hospital admission, hematological diagnosis, pathological diagnosis, and final dermatological diagnosis. All data were analyzed using SPSS version 21. Data are presented as mean ± SD for continuous variables.

## 3. Results

A total of 1391 inpatient referrals were received by the dermatology department during the study period. Overall, 78 (5.6%) were from the hematology department, six of which were rejected by the dermatology department. Almost all referral requests were managed on the same or the following day with only two requests after 3 days. There were more female (n = 40; 51.3%) than male patients (n = 38; 48.7%). The average ± SD age of patients was 40.7 ± 19.8 years (range 15 to 95 years). The majority of patients were Saudi; only five (6.41%) patients were non-Saudi. Patients were diagnosed with a diverse range of hematological diseases as shown in Table 1. Overall, 53.3% of patients were diagnosed with either AML or ALL.

A total of 98 differential diagnoses were made by dermatologists with only 26 being confirmed by skin biopsy as illustrated in Tables 2 and 3. Morbilliform drug eruption was the most common differential diagnosis followed by infection (Table 2). Graft versus host disease was the most common dermatological condition confirmed by skin biopsy (Table 3). The diagnoses were changed in 12 cases after skin biopsy. Multiple types of dermatitis were diagnosed in hematology ward patients including stasis dermatitis and contact dermatitis (Table 2). The source of infection was not specified in most cases of infection and was treated empirically.

## 4. Discussion

Dermatologists have a significant role in delivering care to hospitalized patients even though dermatology has recently become an outpatient service [13]. In particular, hematology inpatients who are diagnosed with malignancy commonly have other cutaneous problems due to the cancer or its treatment, or the complications thereof [10, 11]. In the study conducted by Koh, the total number of referrals made by hematologists was 58 (8.3%) during a period of 6 months [11]. By contrast, in the present study, there were only 78 (5.6%) referrals during a period of 2 years which implies that hematologists in this study were more familiar with a diverse range of dermatological diseases. Consistent with the findings of Bauer and Maroon [8], this study revealed that requests for dermatology consultations are generally managed on the same day. Skin biopsy was an essential tool to guide definitive management [8]. In contrast to previous findings, this study showed that morbilliform drug eruption and graft versus host disease were the most common dermatological conditions. However, Koh reported that the most common dermatological conditions in the hematology ward were dermatitis and infective disorders [11]. In a prospective cross-sectional study conducted by Bukhari et al. in Dammam, the most common cutaneous diseases in outpatient clinics were diffuse alopecia, pallor, and skin dryness [10]. A possible explanation for the different results in this study and the findings of Bukhari et al. might be due to the different settings with more benign hematological diagnoses represented in outpatient clinics investigated by Bukhari et al. compared with hematology ward inpatients with more severe dermatological conditions. In addition, in this study, most of the referral requests from hematology were for acute and serious dermatological conditions in comparison to mainly benign dermatological diagnoses such as skin dryness [10]. Furthermore, hematologists in our study may be more familiar with these conditions and able to treat them without dermatological consultation. Therefore, a prospective study is recommended to derive more accurate conclusions about hematologists' experience of the diagnosis of benign dermatological disorders. Several studies have revealed that in general the referring departments underdiagnose common dermatological conditions [3–6]. According to previous studies, dermatological consultation changed the dermatological diagnosis and treatment in more than 60% of patients, which emphasizes the need to provide education for non-dermatologist in challenging cases [2, 4, 5].

## 5. Limitations

A limitation of this study is the small sample size, and some of the data in the charts was missing similar to other studies. In addition, skin biopsy was not performed for all patients as some refused the procedure or the clinical diagnosis was adequate.

## 6. Conclusion

Various dermatological disorders and cutaneous manifestations are observed in hematology inpatients, the most common of which are morbilliform drug eruption and graft versus host disease. A future study is recommended for a longer period of time with a larger sample size.

## Figures and Tables

**Table 1:** Underlying hematological diagnoses.

**Hematological Diagnosis**	**N (**%**)**
Acute myeloid leukemia	27 (35.1)
Acute lymphoblastic leukemia	14 (18.2)
Hodgkin lymphoma	8 (10.4)
Non-Hodgkin lymphoma	5 (6.5)
Iron deficiency Anemia	5 (6.5)
Non-Langerhans cell histiocytosis	5 (6.5)
Multiple myeloma	4 (5.2)
Chronic lymphoblastic leukemia	2 (2.6)
Langerhans cell histiocytosis	2 (2.6)
Hairy cell leukemia	2 (2.6)
Sickle cell anemia	1 (1.3)
Pancytopenia	1 (1.3)
Others	1 (1.3)

**Table 2:** Differential diagnoses made by dermatologists.

**Differential Diagnosis**	**N (**%**)**
Morbilliform drug eruption	17 (17.3)
Infection	13 (13.3)
Graft versus host disease	8 (8.2)
Toxic erythema of chemotherapy / Neutrophilic eccrine hidradenitis	6 (6.2)
Petechiae and purpura	5 (5.1)
Varicella/herpes zoster	4 (4.1)
Exanthem	4 (4.1)
Leukemia cutis	3 (3.1)
Sweet syndrome	3 (3.1)
Unknown	2 (2.0)
Cellulitis	2 (2.0)
Intertrigo	2 (2.0)
Furunculosis	2 (2.0)
Vasculitis	2 (2.0)
Oral candidiasis	1 (1.0)
Ecthyma gangrenosum	1 (1.0)
Bullous impetigo	1 (1.0)
Kaposi sarcoma	1 (1.0)
Prurigo Simplex	1 (1.0)
Acne	1 (1.0)
Neurofibromatosis	1 (1.0)
Ecchymosis	1 (1.0)
Ecthyma	1 (1.0)
Pseudomonas ulcer	1 (1.0)
CMV^*∗*^ ulcer	1 (1.0)
Lichen Simplex Chronicus	1 (1.0)
Mycosis fungoides	1 (1.0)
Lichen planus pigmentosus	1 (1.0)
Ashy dermatosis	1 (1.0)
Seborrheic keratosis	1 (1.0)
Erythroderma	1 (1.0)
Pressure ulcer	1 (1.0)
Stasis dermatitis	1 (1.0)
Folliculitis	1 (1.0)
Radiation dermatitis	1 (1.0)
Contact dermatitis	1 (1.0)
DRESS^*∗∗*^ syndrome	1 (1.0)
Erythema nodosum	1 (1.0)
Abscess	1 (1.0)

*∗* Cytomegalovirus; *∗∗* drug reaction with eosinophilia and systemic symptoms.

**Table 3:** Dermatological diseases diagnosed in hematology inpatients confirmed by skin biopsy.

**Dermatological condition**	**N (**%**)**
Graft versus host disease	8 (30.8)
Morbilliform drug eruption	3 (11.5)
Unknown	3 (11.5)
Mycosis fungoides	2 (7.7)
Infection	2 (7.7)
Leukemia cutis	1 (3.8)
Varicella ∖ herpes zoster	1 (3.8)
Seborrheic keratosis	1 (3.8)
Histiocytic infiltrate with histiocytic vasculitis	1 (3.8)
Hyperpigmentation	1 (3.8)
Morphea	1 (3.8)
Sweet syndrome	1 (3.8)
Vasculitis	1 (3.8)

## Data Availability

The data used to support the findings of this study are included within the article.

## Abbreviations

ALL: Acute lymphoblastic leukemia
AML: Acute myeloid leukemia
KASCH: King Abdullah Specialist Children Hospital.
